# Improving the Prediction of Survival in Cancer Patients by Using Machine Learning Techniques: Experience of Gene Expression Data: A Narrative Review

**Published:** 2017-02

**Authors:** Azadeh BASHIRI, Marjan GHAZISAEEDI, Reza SAFDARI, Leila SHAHMORADI, Hamide EHTESHAM

**Affiliations:** Dept. of Health Information Management, School of Allied Medical Sciences, Tehran University of Medical Sciences, Tehran, Iran

**Keywords:** Survival, Cancer, Gene expression, Machine-learning techniques, Clinical decision support system

## Abstract

**Background:**

Today, despite the many advances in early detection of diseases, cancer patients have a poor prognosis and the survival rates in them are low. Recently, microarray technologies have been used for gathering thousands data about the gene expression level of cancer cells. These types of data are the main indicators in survival prediction of cancer. This study highlights the improvement of survival prediction based on gene expression data by using machine learning techniques in cancer patients.

**Methods:**

This review article was conducted by searching articles between 2000 to 2016 in scientific databases and e-Journals. We used keywords such as machine learning, gene expression data, survival and cancer.

**Results:**

Studies have shown the high accuracy and effectiveness of gene expression data in comparison with clinical data in survival prediction. Because of bewildering and high volume of such data, studies have highlighted the importance of machine learning algorithms such as Artificial Neural Networks (ANN) to find out the distinctive signatures of gene expression in cancer patients. These algorithms improve the efficiency of probing and analyzing gene expression in cancer profiles for survival prediction of cancer.

**Conclusion:**

By attention to the capabilities of machine learning techniques in proteomics and genomics applications, developing clinical decision support systems based on these methods for analyzing gene expression data can prevent potential errors in survival estimation, provide appropriate and individualized treatments to patients and improve the prognosis of cancers.

## Introduction

Today, cancer is one of the major health problems worldwide (1–3). Cancer research in the 21st century has become one of the most common efforts. The International Agency for Research on Cancer, based on 2002 dataset, estimated that the numbers of cancer patients are 25 million and the American Cancer Society in 2004, announced that officially cancer would replace heart disease as the main cause of death (4–6). Despite the many advances in early detection of diseases, cancer patients have a poor prognosis and survival rate for such patients are low (7–9). Correct perception of the biologic behavior of the tumor and its analysis, help to correct choice of treatment and has a potential to improve the consequences of cancer as well. Accurate estimation of prognosis and survival duration is the most important part of a process of clinical decision-making in patients with malignant disease (9). The first step to making sure that cancer patients have received proper care is to improve the ability of physicians to formulate this type of estimation (10).

The prediction of prognosis includes the vast range of decisions about different aspects of cancer treatment (10). The gene expression profiles obtain from different tissue types (11). By comparing the genes expressed in normal tissue and diseased tissue can bring better insight and understanding of the cancer pathology and help to physicians in decision-making. Checking gene expression patterns for attributes associated with the clinical behavior are very important, because these patterns, examine prognosis and leading to the alternative approach to understand the molecular and physiological mechanisms (10–12).

Gene expression pattern analysis offers ways to improve the diagnosis and classification of risk for many cancers (11, 12). Studies have marked the power of analytical methods than histological and clinical criteria in survival prediction. Recently in artificial intelligence domain, developing clinical decision support systems based on machine-learning methods to analyze gene expression data has facilitated and improved the medical prognosis. Studies have shown the higher accuracy of machine learning algorithms than regression models in predicting cancer survival (12, 13). Gene expression data have the potential to prevent errors caused by fatigue and impatience of oncology experts in survival estimation. Analyzing such a data by using machine-learning techniques leads to developing clinical decision support systems for the correct estimation of survival time and so provides proper treatments to patients according to their survival. This achievement can prevent unnecessary surgical and treatment procedures that increase the use of human resources and time that impose unnecessary costs on patients and the health care system (8, 14, 15).

This study has highlighted the advantages of machine learning techniques in survival prediction of cancer patients based on gene expression data.

## Methods

This review article was conducted by searching articles between 2000 to 2016 in scientific databases (SCOPUS & Pub Med & Google Scholar & IEEE) and e-Journal (science direct), and by using keywords such as machine learning algorithms, gene expression data, survival and cancer. Non-English and unavailable full texts and also the studies that not defined as a journal article were excluded from this study.

## Results

### Microarray Technology and Gene Expression Data

Several genes and proteins with abnormal function and expression contribute to the cancer development and its pathogenesis (9, 16, 17). Gene expression measures the level of gene activity in a tissue and thus gives information about the complex activities of cells. This data usually obtain by measuring the activation and function of genes during their translation. Since cancers are associated with genetic abnormalities, gene expression data can display these abnormalities. To obtain information about these abnormalities, microarray techniques largely have used. Microarray is a technology for measuring the expression and activities of dozens, hundreds or thousands of genes or proteins simultaneously on a small scale, to monitor changes in their structure and activity. The microarray process involves raw data preprocessing to gain gene expression matrix and then analysis it to show the differences and similarities of gene expression (7, 13, 18, 19). The ultimate goal of this technology is discovering a new treatment or reforming previous treatments based on a combination of genetic tumors (11, 20, 21).

Three categories of studies based on microarrays technology are as follows; Class Discovery, Class comparison, and Class prediction. Class Comparison; compare the gene expression profiles of two or more groups of patients. The aim of this category is to find the genes that have different expression levels in groups. Class Discovery; discover sub-groups that are in similar gene-expression profiles. The most common technique in this type of study is clustering. In Class Prediction, categories are predefined and the aim is to determine which class is the target sample belongs to (8, 16, 19).

In recent years, microarray technology has provided great evolution in the biomedical sciences and considered in a vast domain of genomics analysis, such as drug discovery, identifying genes and successful clinical diagnostics. The ultimate goal of this technology in cancer is to discover new genetic markers in the diagnosis, treatment and prediction of patient outcomes based on the genetic components of the tumors (8, 16).

### Survival prediction in patients with cancers

Cancer prognosis is related to three predictive facets that consist of catching cancer, cancer recurrence, and cancer survivability. The first case predicts the likelihood of developing a type of cancers before the disease occurrence. Second case trying to predict the likelihood of redeveloping cancer and the third case predicts outcomes after the diagnosis of the disease such as life expectancy, survivability, progression and tumor drug sensitivity (22).

The survival is an interval that patients are beginning from one starting point until the occurrence of an event, like the period of the beginning and end of a recovery or the time of an illness diagnosis until death (23). Survival analysis is often used to evaluate data from time-to-event in medical research. Often oncologists face the difficult tasks of prognosis and survival in patients with incurable malignancy. Their assessments in these cases are based on clinical experience and comprehensive knowledge of patients. But, such predictions with 20% to 60% accuracy, are largely unreliable, inaccurate and usually more optimistic. Physicians usually tend to over-estimate survival in patients with advanced cancer and sometimes, in spite of the willingness of patients to express prognosis, they refuse to tell it (23, 24). Genetic factors, size, grade and stage of the tumor and the relationship between physicians and patients are vital in survivability prediction. When physicians have a good understanding of their patients’ prognosis, patients likely receive less invasive care (8, 16, 25). Hence, in the medical decision-making process, the ability of physician to formulate a correct estimation of survival among patients with advanced and incurable cancers is necessary (24, 26).

### Machine learning in analysis of gene expression data

The levels of gene expression definitely associated with overall survival of cancer patients (9). There are a high association between gene expression levels and survival and various studies have indicated the power of this type of data than clinical data and other prognostic factors (21, 27). The superiority of gene expression data was demonstrated in providing individualized and right treatments in malignant patients (28, 30–33). Analysis of gene profiles is helpful to improve the accuracy of survival estimation and histopathological classification (28).

Using predictive tools is the important step in this analysis (26). Machine learning methods have the ability to develop survival predict models based on gene expression data (16). Machine learning is a subfield of computer sciences that create and check algorithms to facilitate pattern recognition, classification, and prediction. Supervised and unsupervised learning are two main facets of mechanization in the machine learning that have applications in biology. The examples of supervised machine learning techniques are ANN and Decision Tree and a popular unsupervised learning technique is clustering (34–36).

**Fig. 1:**
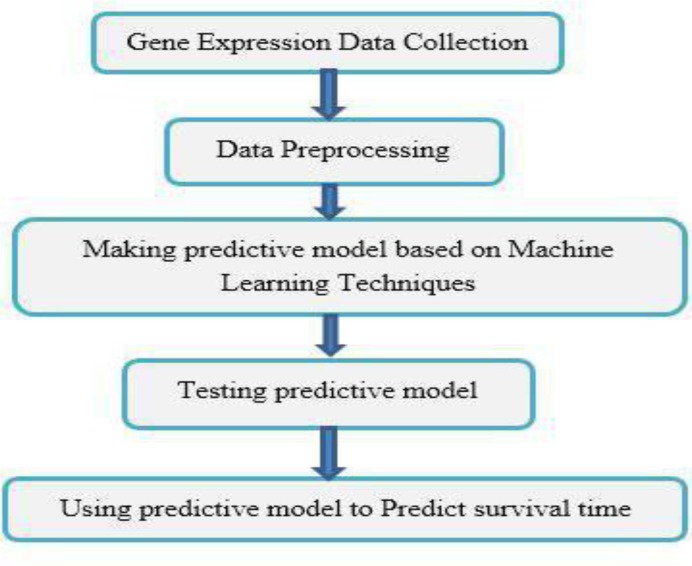
The process of analyzing gene expression data by using machine-learning techniques (9)

In analyzing gene expression by using machine learning techniques, at first preprocessing methods extract and analyze gene expression data. In this step, inputs are identified. Then using machine-learning algorithms, predictive models are built and tested. After, this model based on inputs (gene expression data) can predict outputs (survival time) in cancer patients (8, 9, 28). Fig. 1 briefly indicates this process (9).

Different studies strongly marked the powerful ability of machine-learning techniques to identify patterns, process the interactions of gene expression data and improve the accuracy of cancer prediction, susceptibility, and recurrence (9, 37). Also, these methods can reduce potential errors that cause by fatigue and impatience of oncology experts in survival estimation (37, 38). Predictive models that have created by using these analytical techniques and based on gene expression data can help physicians to optimize clinical decision-making, provide individualized treatment, manage the patients, and reduce the cost puts patients under pressure and the healthcare systems. We have demonstrated each of these studies, cancer type, participants’ number, machine learning techniques have used and the obtained benefit of them (9, 12–14, 18, 21, 22, 27–33, 38–47) (Table 1).

**Table 1:** Experiences of survival prediction by using machine learning methods and based on gene expression data on cancer patients

**Cancer Type**	**Participants number**	**Machine learning technique**	**Training data**	**Benefits**	**Reference**
Mantle Cell Lymphoma (MCL)	N/A	Bayesian Model Averaging (BMA)	Genomic	Analyzing survival with high precision and low cost by using BMA .	Moslemi et al (2016)
Esophageal adenocarcinoma	64	Tail-strength statistic and Cox regression analysis	Genomic	Creating high association between gene expression levels and survival	Pennathur et al (2013)
Esophageal squamous cell carcinoma	12	Clustering	Genomic	Well predicting by using gene expression data than other prognostic factors.	Ishibashi et al (2013)
Non- small cell lung carcinomas	91	Hierarchical clustering	Genomic	Improving histopathological classification	Hou et al (2010)
Diffuse large B-Cell lymphoma (DLBCL)	58	Artificial neural networks	Genomic/Clinical	Creating correct prediction of survival time with high accuracy	Yen-Chen Chen et al (2009)
Astrocytic tumor	65	Artificial neural network	Genomic	Creating a novel model by using ANN for grading Astrocytic tumor	Petalidis et al (2008)
Lung adenocarcinomas	86	Random committee and Bayesian belief networks	Genomic	Providing correct prediction of patient outcomes and individualized treatment and also increase survival time	Guo et al (2006)
Esophageal carcinoma	418	Artificial neural networks	Genomic/Clinical	Providing more accurate prognosis	Sato et al(2005)
Breast carcinomas	295	Decision tree analysis	Genomic/Clinical	Improving cancer classifications, clinical decision making and patients’ treatment	Chang et al (2005)
Malignant pleural mesothelioma	21	Artificial neural networks	Genomic	Improving appropriate therapy	Pass et al (2004)
Hepatocellular carcinoma (HCC)	90	Clustering	Genomic	Providing a source for treatment selection	Lee et al (2004)
Diffuse large B-cell lymphoma	N/A	Clustering techniques	Genomic/Clinical	Providing powerful tool for diagnosing and treating cancer	Bair et al (2004)
Neuroblastoma	49	Artificial Neural Networks	Genomic	Helping physicians in patient management	Wei et al (2004)
Breast cancer	78	Artificial Neural Networks	Genomic	Selecting patients with poor prognosis	Lancashire et al (2003)
Diffuse large B-cell lymphoma	40	Artificial Neural Networks	Genomic/Clinical	Predicting survival time with high accuracy	O’Neil and song (2003)
Lung adenocarcinomas	N/A	Univariate Cox analysis	Genomic	Determining of high-risk groups	Beer et al (2002)
Diffuse large B-cell lymphoma	40	Fuzzy Neural Network	Genomic	Extracting biological markers with high accuracy	Ando et al (2002)

## Discussion

According to the cancer priority as one of the important health issues in the world that imposes mortality and costs, there is an urgent need for survival prediction strategies. One of the main goals in cancer patients is the estimation of survival. It leads to better management, optimal uses of resources and providing individualized treatments (1, 3).

In recent years, the advent of machine learning algorithms to extract and analysis, gene expression in cancer tissues had led to use of these techniques in detection, diagnosis, classification and prediction of cancer patients. Also the researches in biology and medicine domain have confirmed new achievements in cancer prognosis (34–36).

In analyzing gene expression data by using machine-learning techniques for survivability prediction, at first, these data are extracted and analyzed by preprocessing methods. Then, by making and testing predictive models using machine learning algorithms, survival duration can be predicted (8, 28). In this study, we highlighted the advantages of using machine-learning techniques in analyzing gene expression data in order to correct prediction of patients’ survival and histopathological classification. By investigating studies with different kinds of cancers, our findings indicated the power of gene expression data in survival prediction than other prognostic factors such as clinical data. The studies of Ishibashi et al. on patients with esophageal squamous cell carcinoma and Pennathur et al. in esophageal adenocarcinoma supported this point. According to their findings, there is a high association between gene expression levels and survival and also gene expression data provide better prediction of cancer patients (21, 27).

Some studies used the combination of genetic and clinical data in the prediction of survival. Using gene expression data can provide a more accurate prognosis and cancer classifications as well as providing correct decision making for patients’ treatment (9, 28, 31, 40, 44) supported the effectiveness of gene expression data in helping physicians in patient management and providing a source for appropriate treatment of cancer patients (32, 45). Bair et al. highlighted such a data provide powerful tool for diagnosing and treating any type of malignancy (44).

Because of the capabilities of machine-learning techniques in the vast range including detection, diagnosis, classification and prediction, we found the feasibility of such analytical techniques in creating a clinical decision support tool to predict survival in cancer patients (48, 49).

Chang et al. showed the improvement in classifications of breast cancer by using decision tree analysis (31). Other studies mentioned the high precision and low cost of Bayesian Model techniques in survival prediction of Mantle Cell Lymphoma (30). In addition, Guo et al. using the Random committee and Bayesian belief networks on Lung adenocarcinomas showed the capacity of these algorithms in the true prediction of patient outcomes (42).

Among machine learning algorithms, our review of various studies has indicated the frequency usage of (ANN) with high accuracy in survival prediction for any malignancy based on gene expression data (9, 28, 32, 43, 45, 46).

However, the combination of ANN and Fuzzy logic with 93% accuracy is superior and powerful tool for extracting significant biological markers (38, 39).

## Conclusion

Developing clinical decision support systems based on these algorithms for analyzing gene expression data can improve survival prediction and prognosis of cancer patients.

## Ethical considerations

Ethical issues (Including plagiarism, Informed Consent, misconduct, data fabrication and/or falsification, double publication and/or submission, redundancy, etc.) have been completely observed by the authors.
